# Draft Genome Sequence of a Pathogenic O86:H25 Sequence Type 57 *Escherichia coli* Strain Isolated from Poultry and Carrying 12 Acquired Antibiotic Resistance Genes

**DOI:** 10.1128/genomeA.01107-15

**Published:** 2015-09-24

**Authors:** Daniela Jones-Dias, Vera Manageiro, Daniel Ataíde Sampaio, Luís Vieira, Manuela Caniça

**Affiliations:** a National Reference Laboratory of Antibiotic Resistances and Healthcare Associated Infections (NRL-AMR-HAI), Department of Infectious Diseases, National Institute of Health Dr. Ricardo Jorge, Lisbon, Portugal; b Centre for the Studies of Animal Science, Institute of Agrarian and Agri-Food Sciences and Technologies, Oporto University, Oporto, Portugal; c Innovation and Technology Unit, Human Genetics Department, National Institute of Health Dr. Ricardo Jorge, Lisbon, Portugal

## Abstract

*Escherichia coli* is a commensal bacterium that is frequently associated with multidrug-resistant zoonotic and foodborne infections. Here, we report the 5.6-Mbp draft genome sequence of an *E. coli* recovered from poultry, which encodes multiple acquired antibiotic resistance determinants, virulence factors, pathogenicity determinants, and mobile genetic elements.

## GENOME ANNOUNCEMENT

*Escherichia coli* is the most prevalent commensal microorganism of the human and animal gastrointestinal tract, remaining one of the most frequent causes of bacterial infections worldwide (1). Diseases caused by this pathogen are difficult to treat due to the presence of antibiotic resistance determinants that regularly render the isolates multidrug resistant (2). Moreover, antibiotic resistance genes are frequently inserted in mobile elements that facilitate transmission between bacteria (3, 4).

*E. coli* INSLA289 was isolated from broilers belonging to an animal farming facility with unknown clinical history and was recovered from macerates of organs during postmortem examination. It was tested for its antibiotic resistance and found to be nonsusceptible to penicillins, first-, second-, third-, and fourth-generation cephalosporins, aztreonam, quinolones, tetracycline, aminoglycosides, and nitrofurantoin. Genomic DNA was extracted using a DNeasy blood and tissue kit (Qiagen). Libraries were prepared from 1 ng of genomic DNA using the Nextera XT DNA sample preparation kit (Illumina, San Diego, CA) according to the manufacturer’s instructions. Whole-genome sequencing (WGS) was performed using 150-bp paired-end reads on a MiSeq (Illumina). Sequence reads were then trimmed and filtered according to quality criteria and assembled *de novo* using CLC genomics workbench version 8.0 (Qiagen). The NCBI prokaryotic genome automatic annotation pipeline (PGAAP) was used for annotation. PathogenFinder 1.1, ResFinder 2.1, VirulenceFinder 1.4, SerotypeFinder 1.1, and MLST 1.8 were used to estimate pathogenicity determinants, antibiotic resistance genes, virulence factors, multilocus sequence type (MLST), and serotype of this isolate, respectively (5–8).

The draft genome of the *E. coli* INSLA289 was assembled *de novo* into 300 contigs (each >200 bp long), which together comprised 5,584,816 bp. Global results indicated a GC content of 50.6%, an average coverage of 175.2, and an *N*_50_ of 99.169 bp. The largest obtained contig was 444,397 bp, presenting a coverage of 131.7-fold. The obtained contigs were searched against the GenBank database nucleotide collection (nr/nt) using Megablast. Globally, a total of 70 contigs matched multiple plasmid sequences therein deposited.

ResFinder 2.1 (90% identity and 40% minimum length) enabled the detection of 12 antibiotic resistance genes, *bla*_CTX-M-1_ (contig 77), *bla*_SHV-12_ (contig 179), *bla*_TEM-116_ (contig 193), *aadA1y* (contig 20), *aph*(*3′*)*-Ic* (contig 172), *strA* and *strB* (contig 172), *sul2* (contig 72), *dfrA1* (contig 20), *tetA* (contig 188), *tetB* (contig 99), and *sat2* (contig 20). This ST57 *E. coli* isolate also carried an IS*10*-disrupted In*2-4* class 2 integron, where *dfrA1*, *sat2*, and *aadA1y* were accommodated, resulting in the disruption of the *attI2* integration site. Seven virulence factors were also detected, *cma* (contig 154), *ireA* (contig 98), *prfB* (contig 9), *ioN* (contig 89), *tsh* (contig 86), *iss* (contig 89), and *iha* (contig 20). Moreover, the isolate expressed serotype O86:H25 and displayed a prediction of 91.4% for being a human pathogen.

This draft genome sequence constitutes a valuable resource for international genomic comparison studies and may be helpful for identifying genomic traits associated with the zoonotic potential of multidrug-resistant *E. coli* isolates.

### Nucleotide sequence accession numbers.

This whole-genome shotgun project has been deposited at DDBJ/EMBL/GenBank under the accession number LHAT00000000. The version described in this paper is version LHAT01000000.
